# Correction: Relationship between the morphological, mechanical and permeability properties of porous bone scaffolds and the underlying microstructure

**DOI:** 10.1371/journal.pone.0242181

**Published:** 2020-11-05

**Authors:** The PLOS ONE Staff

The following information is missing from the Funding statement: Dr. Liangliang Cheng received the funding from Dalian Science and Technology Innovation Fund Project (grant number: 2020JJ27SN077). The publisher apologizes for the error.
