# Supplementary material for: Relationship between the morphological, mechanical and permeability properties of porous bone scaffolds and the underlying microstructure
Source: PLoS One. 2020 Sep 1;15(9):e0238471. doi: 10.1371/journal.pone.0238471 (PMC7462274; doi:10.1371/journal.pone.0238471)
Supplement: Attachment — Submitted filename: My review.pdf [file pone.0238471.s001.pdf]

The current study investigates the relationship between topology, mechanical properties and permeability of triply periodic minimal surfaces (TPMS) based lattices and other strut-based lattices. The work follows a finite elements analyses framework and experimental validation of the mechanical part.

Unfortunately, this paper is weakly written, and does not provide any new significant findings at all. therefore, it is not recommended for publication.

## Introduction

The introduction seems to be missing lots of relevant works that are hard to miss and reported the properties of TPMS-based materials. For example, the work of Montazerian et al (10.1016/j.matdes.2017.04.009) reported the elastic properties and permeability of a wide range of TPMS-based materials including those reported in this work. The study of Kapfer et al (10.1016/j.biomaterials.2011.06.012) which dates back to 2011 also investigates the elastic and permeability properties. Abueidda et al. (10.1016/j.mechmat.2016.01.004) also reported the elastic properties of TPMS-based materials numerically. These are only few examples.

The authors claim that literature is missing studies that compare the properties of TPMS based materials with other lattice types. This is also not true as the work of Al-Ketan and coworkers (10.1016/j.addma.2017.12.006), (10.1002/adem.201800029), and (10.1557/jmr.2018.1) have extensively discussed the difference in mechanical behavior of TPMS-based materials in comparison with strut-based materials.

## Results and discussion

Unfortunately, the presented results seem to be missing a lot, the authors claim that they performed a mesh convergence study without reporting the results of this study, or the criteria used to decide on the mesh size. The authors also did not show any stress contours!!

The authors also claim to have validated the mechanical properties experimentally. However, the authors did not show any figure of the 3D printed samples, or if the actual relative density matches that of the designed. The authors also did not present a single stress-strain response or the deformation pattern of the different lattices.

On page 16, the authors state “However, it should be noted that, because the TPMS sheet solids have the same microstructure topology as their network solid counterparts, the features of the mechanical and permeability properties of the TPMS sheet solids should be similar to their network solid counterparts. However, this needs to be confirmed in the future studies.”

This statement is very wrong and the properties should not be similar. In fact, several studies have already shown that solid-networks and sheet-networks have very dissimilar properties. For example, Kapfer et al (10.1016/j.biomaterials.2011.06.012) and Al-Ketan et al (10.1002/adem.201800029) among others. In fact, a recent review by Al-Ketan et al ( 10.1002/adem.201900524) discusses in detail the difference between sheet-based and network-based lattices.

In conclusion, this work is significantly missing a lot of proper analysis, data presentation, and comprehensive discussion in light of the ubiquitous studies presented to data with respect to TPMS-based materials. this reviewer does not recommend this work for publication.
