# Supplementary material for: Relationship between the morphological, mechanical and permeability properties of porous bone scaffolds and the underlying microstructure
Source: PLoS One. 2020 Sep 1;15(9):e0238471. doi: 10.1371/journal.pone.0238471 (PMC7462274; doi:10.1371/journal.pone.0238471)
Supplement: Attachment — Submitted filename: Response to Reviewers.docx [file pone.0238471.s003.docx]

# Replies to reviewers’ comments

**Manuscript Number:** PONE-D-20-13348R1

**Title:** Relationship between the morphological, mechanical and permeability properties of porous bone scaffolds and the underlying microstructure

Thanks to the reviewers for their valuable suggestions. They have contributed to improve the quality of the paper. We hope the responses we provide below will answer their concerns and shed light on the unclear parts of the study.

**Reviewer #3:**

It is clear that the authors have made substantial changes to their manuscript based on the questions and comments of several reviewers. I stand by my original comment that the paper presents results which are both novel and useful, especially for the designer of scaffold structures for a range of mechanical and fluid-flow applications.

But the authors have not addressed my chief criticism from the first review; the issue of the quadratic fits for compressive modulus and shear modulus. I originally stated that these fits are not generally used; the Gibson-Ashby power laws are used instead because they have a basis in the mechanical deformation of cellular struts. I asked the authors to justify their use of quadratic fits, which they have not done. By 'justify' I mean demonstrate that the fitting function is valid for this data type by considering the underlying phenomena. The authors should either provide a valid justification for all choices of fitting functions in the manuscript, or remove those which cannot be justified.

**Replies:** Sorry, we did not address the issue properly. Following the suggestion, we have removed the quadratic fitting between the mechanical properties and the porosity and instead focus on the use of Gibson-Ashby power law to explain the underlying mechanism. Please see the updated Table 4 and the relevant update in the manuscript.

“Regarding the mechanical property, to reflect the underlying physical phenomena, the relationship between the relative elastic compressive modulus and the scaffold volume fraction were described using the exponential function proposed by Gibson and Ashby…”(Page 8 Lines 23 - 26)

“Regarding the scaffold permeability and the surface-to-volume ratio, the statistical regression equations (quadratic or other forms) and the coefficient of determinations (R^2^) were computed for the relationships between them and the scaffold porosity. The reasons for deriving these statistical regression equations are to enable the interpolation of the data points to the full scaffold porosity range and to facilitate the scaffold design by using these relations.” (Page 9 Lines 7-12)

Regarding figure 6(a), why is it that, for each scaffold type, the surface-to-volume ratio barely changes over the examined range of porosity? Surely the S/V should be far greater when the porosity takes a large value; i.e. when there is a lot of surface but very little volume. Can the authors explain the 'hump shaped' curves of 6(a)?

**Replies:** Thanks for the suggestion. We now added the data on the relationship between the surface-to-volume ratio and the porosity in the full range of scaffold porosity (0.0 to 1.0) (Fig. 6a). From the added figure, it is clearly shown that the surface-to-volume ratio barely changes with the porosity is not true. The reason we presented Fig. 6b is that some scaffolds will lose the connectivity when the porosity gets too big. Therefore, we chose the range (i.e., 0.3 – 0.7) in which all the scaffolds are valid and made the comparison in these meaningful range. We believe the reason for the ‘hump shaped’ curves is that: the inner surface area was used to calculate the surface-to-volume ratio (when the porosity gets lower, there are more overlapped surfaces; when the porosity gets higher, the inner surfaces get fewer), and so the surface-to-volume ratio is not a monotonic function of the scaffold porosity. Please see the updated Figures 6 and 7, and the clarification in the manuscript as below:

“For all the scaffolds except the Octa-based one, the surface-to-volume ratio is not a monotonic function of the porosity and the surface-to-volume ratios are the highest when the porosity is 0.5, and start to decrease when the porosity is away from 0.5, the reason for which could be that the overlapped inner surfaces increase when the porosities get lower and there are fewer inner surfaces with the increase of the scaffold porosity.” (Page 11 Lines 15-20)

“The interpolated values using the fitted quadratic relationships and the comparison of the surface-to-volume ratio in the porosity range from 0.3 to 0.7 are presented in **Fig 6b**.” (Page 11 Lines 24-26)

As a final note, the authors should consider re-writing the conclusion section, as it is not appropriate to simply have the results repeated there as bullet points. Rather, the overarching findings of the paper should be clearly summarized and placed into context.

**Replies:** Thanks for the suggestion. We have re-writing the conclusion section as below:

- “The bending dominated scaffolds (e.g., Diamond, Gyroid, Schwarz P, Fischer-Koch S and R-RD) tend to have a higher effective shear modulus. The stretching dominated scaffolds (e.g. Schwarz P, Cube, FD-Cube and Octa) tend to have a higher effective compressive modulus.
- The relative shear modulus of the scaffold changes faster than the relative compressive modulus, i.e., when the same amount of change in the scaffold porosity is made, the corresponding change in the relative shear modulus is larger than that in the relative compressive modulus.
- The permeability of the scaffold depends on the arrangement of the underlying microstructure, e.g., the structures with the simple and straight pores (e.g., Cube) have a higher permeability than the structures with the complex pores (e.g., Fischer-Koch S). ”

All the changes in the manuscript were highlighted in yellow to help the reviewers track the changes. We hope we have clearly answered all the reviewers’ concerns. If there is still something unclear, please feel free to contact me by email.

Sincerely,

Yongtao

Yongtao Lu, Ph.D

Department of Engineering Mechanics

Dalian University of Technology, Dalian

No.2 Linggong Road,

116024, Dalian, China

Email: [yongtaolu@dlut.edu.cn](mailto:yongtaolu@dlut.edu.cn); [yongtaolu@hotmail.com](mailto:yongtaolu@hotmail.com)
